# Hernia

**Published:** 1829-02

**Authors:** 


### HOSPITAL REPORTS,

*(Principally condensed from various Periodical Publications.)*

### HERNIA.

*Case of Strangulated Crural Hernia, which, after Gangrene had supervened, was cured by an artificial Opening of the Tumor.* By Dr. J. J. Caffort, Surgeon of the Hôtel Dieu at Narbonne.

There are many cases on record of strangulated hernia with gangrene, which were spontaneously cured by the rupture of the sac and intestine, followed by the discharge of the feces through the opening; and some cases are related in which the opening closed of its own accord, after a longer or shorter continuance. Professor Scarpa, who attentively studied the *modus sanandi* in such cases, was of opinion that, in similar circumstances, we ought always to imitate this process which nature sometimes adopts, by opening the hernial tumor with the knife, and cutting away the gut itself, should the exigency of the case require it. Notwithstanding so great an authority, and several facts which tend to establish the soundness of his advice, some surgeons still hesitate, and are afraid to adopt this method. Since, however, this is the only means by which, in desperate cases, we may hope to snatch our patients from almost certain death, all timidity is culpable, and we ought to operate without the least hesitation.

The following case, while it confirms the utility of this practice, presents some striking particulars.

Paul Pech, gardener, æt. twenty-eight, of a robust constitution, was affected for about two years with crural hernia on the left side, which was become irreducible. Notwithstanding this, as it was rather small, Pech was scarcely incommoded by it, and would never submit to the trouble of wearing a bandage.

On the 4th April, 1825, after eating and drinking to excess, and dancing all night, he felt an acute pain in the hernia, which in a short time became extremely voluminous. Symptoms of strangulation having supervened, the village surgeon was called in. Although the patient referred the pain to the hernia, he believed the disorder to be common gastro-enteritis, and ordered leeches to be applied to the epigastrium. As they did not assuage the pain, and as the symptoms of the disease assumed a more formidable aspect, a physician of Narbonne was sent for. He immediately recognised the complaint, and recommended that a surgeon should be brought to the patient as soon as possible. This prudent advice was not adopted till two days afterwards, when the surgeon who came to see the patient finding that all pain had subsided, and that the pulse was small, and perceiving besides all the symptoms which usually indicate actual gangrene, did not venture an operation, which he believed could not be practised with success, and preferred leaving the patient to the care of nature alone.

Pech's state continued to grow worse up to the 14th of April, when all the symptoms became rather milder, and he passed one scanty dejection. The improvement he was experiencing being noticed, he was carried on the 15th to the hospital of Narbonne. M. Cafford found him, on his arrival, in the following state: His countenance anxious; his tongue red at the sides and black in the middle; his abdomen tense and painful; in the left groin a voluminous, hard, round tumor, the circumference of which was red, and the centre rather black, in which part fluctuation was then apparent.

From these symptoms M. Cafford concluded that the gangrene was arrested, and that an inflammatory process for the separation of the dead parts had already commenced; and the reappearance of the stools since yesterday, and the cessation of vomiting, led him to presume that the omentum alone formed the hernia. He thought it would be proper to assist the operations of nature by opening the tumor transversely. When the incision was made through the skin into the sac, a small quantity of pus flowed forth, and he perceived a dark-coloured substance, which, upon inspection, was found to be the omentum in a state of gangrene, and very fetid. Being unable to disentangle it, he was obliged to cut out the greater part. A simple pledget of lint was afterwards applied to the wound, and, as the inflammatory redness was very considerable, a poultice was laid over the whole.

On the 16th, when the dressings were removed, a great quantity of serum issued forth from the wound, and the redness of the contiguous parts was diminished. Another piece of omentum was cut away. Although the local disease was sensibly lessened, Pech was still affected with much fever, and was very thirsty; he had passed no stool, and the tenderness of his abdomen was increased. Yet all these symptoms appeared attributable to his having taken food, contrary to orders. He is now, therefore, only allowed rice water for food.

17th.—The bottom of the wound being exposed, a very large lumbricus was found there, the presence of which removed all doubt as to the perforation of the intestine. This day the redness along the spine of the ilia reappeared: it existed when the patient first entered the hospital, but yesterday it appeared to be quite removed by the poultice. The sort of serum which comes through the wound is so abundant that, during the intervals of dressing it, the bed was completely soaked by that fluid; and, on pressing the abdomen, it spirted out with considerable force; and was so extremely acrid, that it greatly irritated the edges of the wound, and produced erysipelatous inflammation of the scrotum. There is an improvement in the general symptoms, and the bowels have been moved by a clyster.

Up to the 21st, the patient became daily better, barring the redness of the scrotum and over the spine of the ilia, which has considerably increased, and in the former situation two ulcerations have developed themselves. The patient was gradually allowed to partake of vegetable soups, and finally too of those made from meat. During all this time some portion of the food was found on the dressings when the wound was examined. The serum, although still abundant, is, however, much diminished. His bowels are kept open by the use of clysters.

On the 23d, M. Caffort perceived a distinct fluctuation on a level with the anterior third of the spine of the ilia, into which he made an opening, whence a small quantity of pus escaped. Meanwhile the wound in the groin, now much smaller, discharged a still less quantity of serum. The patient's appetite and digestion are good, and scarcely any food escapes through the wound. The opening Dr. C. had made into the abscess which was near the spine of the ilia became enlarged, and a piece of omentum, above four inches long, and twisted like a cord, was discharged through it on the 25th. That it was really omentum, Dr. C. was at first himself inclined to doubt, till he had clearly ascertained the fact by unfolding it with care in the presence of several surgeons, who were equally surprised. From that instant all the wounds began to heal: those of the scrotum and ilia were quite cicatrised by the beginning of May, and on the 28th of the same month the patient left the hospital quite well, with the exception that there remained still in the centre of the cicatrix of the groin an almost imperceptible aperture, that continued to exhale a serous fluid, which slightly soiled the shirt.

Some time after quitting the hospital, the patient having, contrary to Dr. C.'s advice, indulged himself in eating too much at once, the cicatrix burst, and the food was again discharged through the wound; but this accident was speedily remedied by repose and strict attention to diet. He has since married, and has not experienced any return of pain in the part.

This case shows how important it is to open the sac as well as the intestine in cases of hernia with gangrene, either as a substitute for the inflammatory process, (which sometimes fails,) or to promote its effects when it has begun to develop itself. Many similar cases might be cited in support of this opinion, but the one just related presents a most singular fact, viz. the evacuation of a large piece of omentum by an abscess near the spine of the ilia. Why was not this portion of omentum expelled through the wound in the groin? Dr. C. conceives that, when he opened the sac, the omentum was already incarcerated under the spine of the ilia, to which he attributes both his inability to disentangle the omentum there, and the difficulty with which he discovered the perforation of the gut. After he had cut out the portion of omentum which the sac contained, the remainder continued its course, until it finally made its exit through the abscess at the spine of the ilia.

The vast quantity of serum (at least a quart) which was discharged during the intervals of dressing the wound, is a very interesting fact. The slightest exertion on the part of the patient rendered this discharge still more copious. It evidently issued from the cavity of the peritoneum, and if there had been here, as in all known cases, a complete adherence between the omentum, the intestine, and the margins of the hernial opening, this serum would have inevitably caused, by its acrimony and abundance, a most intense peritonitis, and probably death itself.

